# Reconfigurable Digital Satellite-Borne Base Station Design and Virtual Function Fast Migration Algorithm

**DOI:** 10.3390/s23177591

**Published:** 2023-09-01

**Authors:** Yuanji Shi, Ping Wang, Xiaorong Zhu, Hongbo Zhu

**Affiliations:** College of Telecommunications and Information Engineering, Nanjing University of Posts and Telecommunications, Nanjing 210003, China; shiyuanji@nj.ict.ac.cn (Y.S.); 1222014239@njupt.edu.cn (P.W.); zhuhb@njupt.edu.cn (H.Z.)

**Keywords:** virtual function, fast migration algorithm, satellite-borne

## Abstract

A breakthrough in the technology for virtualizing satellite-borne networks and computing and storage resources can significantly increase the processing capacity and resource utilization efficiency of satellite-borne base stations in response to the development trend in multi-star and multi-system converged satellite internet iterative systems. The protocol processing function of traditional satellite communication systems is generally placed in the ground station system for processing, with poor flexibility and low efficiency. As a result, a reconfigurable digital satellite-borne base station architecture design is suggested, allowing for separation of the hardware and software of the satellite-borne base station and flexible programming and dynamic loading of the satellite-borne base station’s functions by software. Meanwhile, a fast adaptive migration algorithm based on multi-dimensional environment awareness is proposed on top of the reconfigurable digital base station, and migration precomputation and real-time computation are added in order to realize rapid deployment of the digital base station system network. Simulation results demonstrate the effectiveness of the proposed algorithm in enhancing system stability and decreasing real-time calculation costs associated with system network migration under conditions of high dynamic changes for each network element in a star-loaded environment. In conclusion, a digital satellite-borne base station system that effectively addresses the issues of low flexibility and high dynamic changes of nodes in the resource-constrained satellite environment can be created by combining the adaptive migration algorithm and the reconfigurable digitized satellite-borne base station architecture.

## 1. Introduction

Satellite internet systems, which can offer low-latency, high-bandwidth, seamless global coverage and flexible and convenient internet access services for users worldwide, are a crucial component of the national space information infrastructure and one of the main focal points of current space competition among major powers.

In recent years, satellite internet systems have often adopted Low Earth Orbit (LEO) satellite constellations. LEO satellites make it possible to achieve low latency and high-bandwidth communication. To achieve low latency, the location that processes the protocol is the key point. If the protocol is processed on the ground, a satellite terminal will opt to send a message to another one. First, it will send the message to the satellite that will forward it to the ground station. Then, after the message is processed by the ground station, it will send the message to a satellite that will then forward it to the destination satellite terminal. But, if processing on the satellite, the communication link between two satellite terminals only includes paths from the satellite terminal to the receiving satellite, and from that satellite to the satellite terminal, and hence the latency should be lower. However, processing on the satellite still poses a problem. Because of limitations to the satellite's resources, it is hard to fulfill all of the processing functions on the satellite. However, if there is an architecture that enables distributed deployment of these functions on different, nearby satellites, satellite-borne processing should be possible. Therefore, in this paper, a design for that architecture is proposed to allow distributed deployment of the processing function on different nearby satellites to process the satellite protocol.

The protocol processing function is typically handled by a ground station system; on-satellite protocol processing, which primarily uses on-satellite transparent forwarding [1], is relatively constrained in traditional satellite mobile communication systems. In current satellite systems, most of the processing functions have been handled by ground station systems. A. M. Marziani performed an analysis of the architecture of the MEO satellite system [2], and it showed that almost all of the processing functions were performed by ground station systems. Recently, 3GPP organizations proposed the NTN network, the processing functions of which are also performed by the ground station system, as described by F. Völk, showing the results of a field trial of a 5G non-terrestrial network [3]. All of these results show that the proposed NTN network experiences long transmission delays and long deployment times, and it is complicated to deploy system functions. So, it is necessary to propose a new architecture to overcome these disadvantages. Recently, a satellite–ground integrated network architecture for 6G was proposed [4]; it deploys the user access functions and lightweight core network functions on the satellite. Additionally, more and more works have focus on processing on the satellite side. T. Kellermann proposed a novel architecture for cellular IoT in future non-terrestrial networks that also put the eNB/gNB and MME on an LEO satellite [5]. However, all these works only propose possible architectures and do not mention how to realize them under the limitations of LEO satellite constellations. Hence, with the development of satellite internet, how to migrate the access network functions from a conventional ground station system up to a satellite has been a hot topic for research in the field of satellite communications. As described in Table 1, the architecture proposed in this paper to fix the problem will have better performance in terms of transmission delays and deployment times compared to other architectures.

Traditional satellite communication ground station systems [6] are implemented in a chimney-like, static manner, using specialized hardware and software, and they have the following flaws and restrictions: First, poor flexibility, which cannot be expanded according to business requirements when new satellites or constellations are launched; second, the traditional ground station lacks openness, as most of the equipment employs private hardware and software interfaces, and the ground station hardware system is strongly coupled with specialized proprietary software that limits innovations to and the evolution of the system; in addition, the closed technology leads to long cycles of service interruption and low operational efficiency [7]; third, because traditional ground stations only perform one function and each set of equipment can only be used with a specific constellation’s technical system, different technology regimes require multiple ground stations, so the traditional ground station architecture is complicated and expensive to build, operate, and maintain [8]. Therefore, when traditional ground station technologies are adopted, it is hard to migrate the access network functions from the ground station systems up to the satellites.

Since the advent of virtualization technology, software-defined networking (SDN) [9,10,11] has been used in the ICT sector to implement network operations and enable software system changes without the frequent replacement of hardware. The base station system must incorporate virtualization technology into the design process for the development of the next-generation satellite internet system [12], then gradually transition to a software-centric network design approach. We should decouple hardware and software to realize the satellite-borne base station [13] functions through software definition, provide satellite-borne base station services “on demand”, and increase system flexibility. Then, it will be possible for us to migrate the ground station functions up to the satellites.

In this paper, we build a multi-star joint digital base station system based on the idea of virtualization and software definition, which realizes RF digitization at the satellite antenna and implements RF front-end digital signal processing, baseband protocol software processing, and high-level protocol processing on the virtualized platform. The digital base station system realizes the decoupling of the base station's hardware and software, so the functions of the base station can be flexibly orchestrated and dynamically loaded by software. But, this also leads to another question, that is, how to deploy the network functions on the satellites quickly to avoid affecting the network performance when the nearby satellites move fast and the topology changes fast. Therefore, we propose a fast adaptive migration algorithm of network functions based on multi-dimensional environment awareness, adding the migration precomputation and real-time computation to build a fast architecture for digitized satellite-borne base station system networks and realize network function migration [14,15,16] while guaranteeing network load balancing and reducing the real-time computing overhead of the system network migration.

To address the issues discussed above, we first adopt the idea of virtualization and software definition to construct a multi-star joint digital base station system architecture. Then, a fast adaptive migration algorithm of network functions based on multi-dimensional environment awareness is proposed to find the proper satellite to migrate the functions to and reduce the migration time on this architecture. Our main contributions are as follows:

- We construct a multi-star joint digital base station system architecture. Since the virtualization technology is used, the hardware and software of the base station are decoupled. Additionally, the functions of the base station can be flexibly orchestrated and dynamically loaded by software, so that they can be distributed and deployed on different nearby satellites.
- Because of the distributed deployment of the functions of the base station, the frequently changing topology of LEO satellites leads to frequent function migration between the satellites. Then, a fast adaptive migration algorithm of network functions based on multi-dimensional environment awareness is proposed to guarantee the network load balance, find the proper satellite to which functions will be migrated, and reduce the real-time computing overhead of the system network migration. Additionally, a reasonable VNF to be migrated is selected by the RAIL resource-aware algorithm, and a multi-objective decision is made by the TOPSIS multi-objective decision algorithm to reduce additional delays brought by migration and the impact brought by migration for the destination node.
- Extensive experiments based on the proposed architecture were carried out to illustrate that the suggested digitized satellite base station system increases the adaptability of satellite network services and enables rapid network deployment, flexible scheduling, and dynamic function loading and that our proposed algorithm can achieve better performance than other algorithms.

The remainder of this paper is structured as follows: The associated work is summarized in Section 2. Section 3 presents a multi-star joint digital base station system architecture. Section 4 describes the suggested multidimensional environment awareness-based quick adaptive migration technique for the architecture. The evaluation results are shown in Section 5. Section 6 concludes this paper.

## 2. Related Research

Some international companies conducted the exploration of wireless network clouds early in the field of terrestrial mobile communications. Through the Domain 2.0 project, AT&T began to reevaluate its business strategy and operational structure about 10 years ago [17], but eventually fell short of its intended objective of “converting AT&T into a software service provider”. However, its network construction O&M concept is worth learning from, and key words such as “open”, “intelligent”, “decoupling”, and “network interoperability “ have been used with high frequency by operators building networks. In 2016, Facebook, Intel, Nokia, Deutsche Telekom and SKT jointly established the TIP project, whose mission is to decouple software and hardware.

In terms of cloud-based wireless network solutions, in 2020 the US company Parallel Wireless announced the launch of an Open RAN-based solution in cooperation with Vodafone Ireland [17], which to a certain extent alleviates the industry’s concerns about operators’ lack of open interface development, IT solution integration and other necessary capabilities, including those of vCU/vDU and Open RAN Controller, which can integrate 2G/3G/4G/5G functional software and support SON and unified orchestration.

International companies are already developing software-defined virtualized ground station systems for satellite communications. At MWC 2019, the European Space Agency’s (ESA) SATis5 satellite-ground integration project demonstrated the combination of satellites and 5G using SDN/NFV and edge computing technologies. The new ground station platform, a virtualized product family that applies a software-defined strategy to ground stations, was unveiled by the American business Kratos on 20 October 2020. With its unique properties of being standardized, open, reconfigurable, scalable, and evolvable, Kratos’ cloud-based ground station architecture enables the maximum possible decoupling between software and hardware, allowing it to continuously adjust its software capabilities to mission requirements. In addition, Papa A suggests a satellite network management system architecture based on SDN for the control demand of 5G satellite–terrestrial converged networks [18], which loads the network’s control logic onto the satellite’s SDN, optimizes the satellite network’s control delay by optimizing the controller’s dynamic deployment scheme, and increases the bearer plane’s flexibility. Y. Liu proposed a shared satellite ground station using virtualization technology [19], and it shows great benefits compared to traditional ground stations. But it is just fit for ground stations and does not consider the limitations of LEO satellite constellations. However, there is little research on how to use virtualization technology to implement reconfigurable virtualization stations on satellite platforms. 

The research on virtualized reconfigurable technology has been mainly conducted for 5G base stations in China. China Mobile put forth a C-RAN wireless cloud network architecture for 5G at the end of 2016. With the introduction of the NFV architecture, processing resources can now be allocated on demand at the “resource pool” level to achieve multiplexing and sharing of processing resources. C-RAN provides a two-level protocol architecture based on centralized/distributed units (CU/DU) [20] on which wireless clouding can be realized. The core of this architecture is the abstraction of processing resources to decouple resources from applications.

Openness on the RAN side is gaining industry acceptance in the 5G era. The O-RAN Test and Integration Center (OTIC) was established by China Mobile, China Telecom, and China Unicom in November 2019 with the goal of advancing the creation and use of 5G small base stations based on open platforms and integrating new IT industry technologies into radio access networks with the goal of assisting operators in the construction of open, agile, intelligent, and flexible end-to-end networks. At Mobile World Congress 2019, China Mobile, together with Lenovo, Intel and Baicells, exhibited 4G/5G O-RAN dual-mode cloud-based white-box small station solutions, and CertusNet released 5G independent grouping network base station products that conform to the O-RAN architecture.

In the investigation of the underlying virtualization technology for 5G base stations, the Institute of Computing, Chinese Academy of Sciences, has proposed a super base station design to address the issue of excessive energy consumption of centralized baseband processing pools. The design is capable of allocating processing resources in accordance with the actual service load, demand, and other realistic scenarios, as well as running tasks on various hardware processing devices in accordance with the real-time requirements of the base station processing tasks.

The virtualization of 5G access networks has been partially studied by the terrestrial mobile communication sector, but more research needs to be performed on RF digitization, baseband virtualization, and integrated resource scheduling of network computing and storage. Additionally, because of its architecture’s focus on terrestrial mobile communications, it is still unable to handle multi-protocol system reconfigurability and multi-star and multi-station resource multiplexing for high- and low-orbit, wide- and narrow-band convergent satellite internet systems. At present, most of the research is focused on the design of virtual station architectures in the field of terrestrial mobile communication. The design of satellite communication systems has not been considered. Moreover, satellite-borne architecture designs have no research foundation.

In the field of satellite communications, little research has been conducted in China on satellite ground stations oriented to rapid functional reconfiguration and on algorithms for fast migration of virtual functions, and there are no mature products related to virtualized satellite measurement, operation and control stations and communication base stations. In this paper, we provide an architecture for a horizontal shared virtual network that converges computing, communication, and storage facing to the complex and dynamic environment on the satellite. In order to realize quick migration of network functions, reduce the real-time calculation overhead of network migration, and achieve balanced network loads, we offer a fast adaptive migration algorithm for network functions based on multi-dimensional environment awareness.

## 3. Design of a Digital Satellite-Borne Base Station for Quick Functional Reconfiguration

The horizontal shared network architecture used by the digital satellite-borne base station is a new access network architecture with physical centralization, logical wide-area distribution, multi-point cooperative transmission, and intelligent dynamic sharing of resources. It combines computing, communication, and storage in one network. In order to achieve cost savings (statistical resource reuse), lower energy consumption, and network optimization, the architecture may dynamically construct the logical network of each network system needed for services in real time and runtime global optimization of the network.

**Figure 1:**
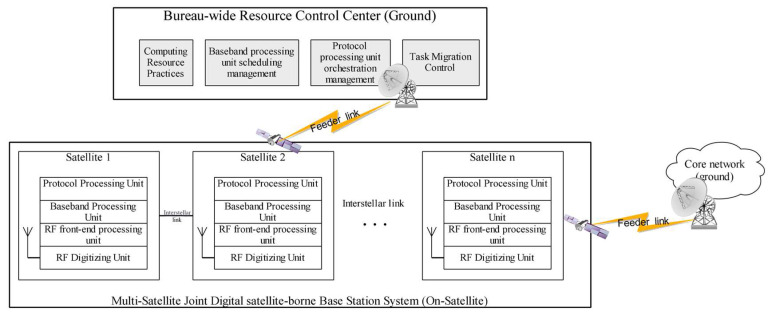
Physical architecture of digital satellite-borne base station system.

**Figure 2:**
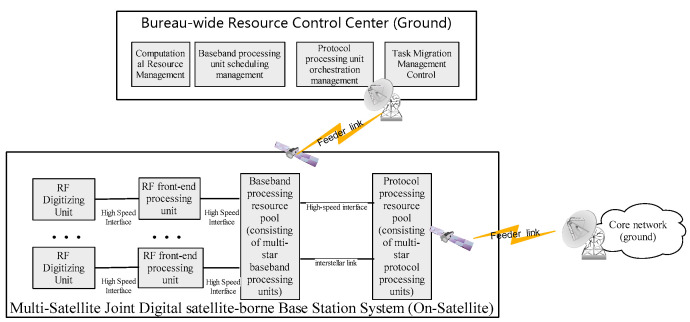
Logical architecture of digital satellite-borne base station system.

Figure 1 depicts the physical connectivity of the digital satellite-borne base station system. The system is made up of several satellites, each of which has an RF digitizing unit, an RF front-end processing unit, a baseband processing unit, and a protocol processing unit. High-speed inter-satellite links are used to exchange data between the satellites. Baseband processing units on numerous satellites constitute a baseband processing resource pool, and protocol processing units on many satellites form a protocol processing resource pool. Both of these processing units are managed by the global resource control center, which is on the ground. The logical connection of the digital satellite-borne base station system, as shown in Figure 2, consists of a multi-star, multi-system protocol processing resource pool, baseband processing resource pool, RF front-end processing unit, and RF digitizing unit to complete the signal and information processing architecture of the whole station. Among them, the protocol processing resource pool is realized through virtualization technology, which is composed of multi-star protocol processing units, and the protocol processing function of each star is programmed and loaded by the global resource control center. The baseband processing units have quick functional reconfigurability to enable waveform software dynamic loading. The baseband processing units of various stars come together to form a baseband processing resource pool. Additionally, the global resource control center schedules and loads the particular baseband processing function of each star. The administration of satellite-borne computing resources, the scheduling management of baseband processing units, the scheduling management of protocol processing units, and the task migration control are all handled by the global resource control center on the ground.

The infrastructural capabilities in this system are controlled and abstracted in a uniform manner, then dynamically allocated to virtual beams and channels as needed through the system’s unified centralized resource control. Using the open interfaces offered by the digital satellite-borne base station system, the global resource control center can dynamically add, configure, manage, monitor, upgrade, and expand virtual beam and channel devices, configure wireless resource management algorithms, and carry out network optimization without being aware of the precise location and physical configuration of the actual physical entities (baseband processing resources, protocol processing resources, etc.).

**Figure 3:**
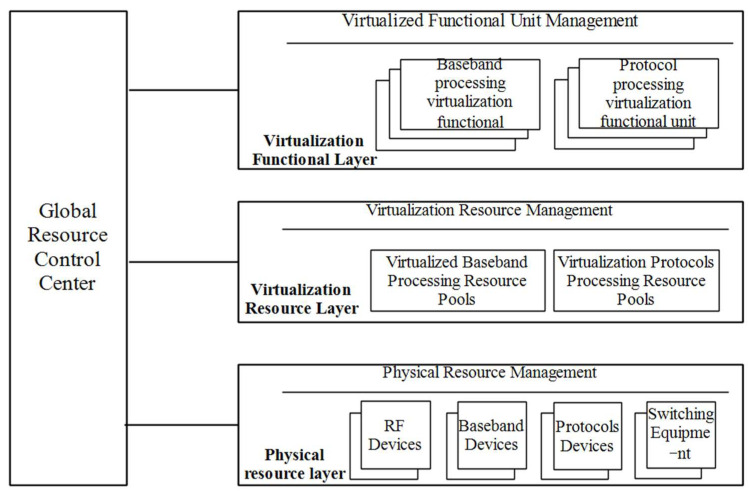
Resource virtualization model of digital satellite-borne base station system.

According to the characteristics of multi-star and multi-protocol information transmission network processing resources, the three-layer virtualization model of a digital satellite-borne base station architecture is defined as shown in Figure 3.

The digital satellite-borne base station architecture network resource virtualization model is divided into three logical layers, which are the physical resource layer, virtualized resource layer, and virtualized function layer. All three layers entities are managed by the global resource control center.

Among them, the physical resource layer is located at the lowermost layer of the digitized satellite-borne base station system and consists of various infrastructures. The infrastructure includes baseband processing equipment, protocol processing equipment, RF front-end processing equipment, RF digitizing equipment, etc. Each device consists of corresponding processors, memory, accelerator, I/O interfaces, etc. The devices on the same satellite are interconnected by wired high-speed links, and the devices across satellites can be interconnected by high-speed inter-satellite links. Each device has a certain processing capacity, i.e., the maximum number of tasks that can be handled by a full load. The processing capacity is quantified by dividing it according to the characteristics of satellite communication resource processing: for example, how many subcarriers of bandwidth are handled as the basic unit. The finer the granularity of capacity division, the greater the management overhead that may result.

The virtualization resource layer focuses on resource virtualization and pooling, how a subset of virtual resources are allocated, the mapping relationships between virtual and physical resources, resource migration, and how to provide a virtual resource view upwards. Virtualized resource management is one of the key factors to realize efficient utilization of each virtualized and pooled resource of a digital satellite-borne base station system.

The virtualized functional layer sits above the virtualized resource layer and primarily enables programmable and dynamically customizable management of virtualized functional units while facing the multi-star multi-protocol transport network. The virtualization function layer applies for corresponding virtual resources from the virtual resource pool view according to the service requirements, and forms protocol-specific processing virtualization function units and baseband processing virtualization function units by dynamically combining and binding the frequency and bandwidth resources, baseband processing resources, protocol processing resources and management control resources required for processing tasks.

The three-layer virtualization model of the digital satellite-borne base station architecture is abstracted in layers that serve each other. The physical resource layer masks physical devices to the upper layers and abstracts the infrastructure as a service for the virtualized resource layer. The physical resource layer abstracts virtualized resources to the physical resource view. The virtualization resource layer, in turn, aggregates the virtualization resources abstracted from different physical devices and provides a virtualization resource view in the form of a new resource pool that is used by the virtualization functional layer. The virtualization function layer then obtains different resources from the virtualization resource pool view to combine into logically independent virtualization function units. With three-layer virtualization, the digital satellite-borne base station system only needs to concentrate on the transparent use of resources, focusing on network services and network optimization, such as the deployment of network optimization algorithms. This eliminates the need to pay attention to the type and location of the physical infrastructure entirely.

From the model and architecture of the digital satellite-borne base station system resource virtualization, it can be seen that the resource virtualization technology is essentially a resource control technology that provides more flexible resource organization and management by allowing the software/hardware resources of the digital satellite-borne base station system to be managed and controlled in different levels and ways, shielding the form of physical facilities for upper-layer applications, decoupling the binding between different physical devices, and providing more flexible resource organization and management methods. The structure of digital satellite-borne base station systems for multi-star multi-protocol regime information transmission networks is made more flexible by the provision of infrastructure and processing capabilities in the form of services to the upper layer of management entities.

In order to realize the fast deployment of a digital satellite-borne base station system network, ensure real-time network migration and reduce the real-time computational overhead of network migration, this paper proposes a fast adaptive network function migration algorithm, based on multi-dimensional environment awareness, that performs network function migration and achieves network load balancing through algorithms for reasonable selection of VNFs and the destination nodes to be migrated.

## 4. A Fast Adaptive Migration Technique Based on Multidimensional Environment Awareness for Virtual Network Functionalities

The digital satellite-borne base station architecture deploys network functions that are on the original dedicated device on top of the common device on the satellite through virtualization technology and cloud computing to improve the flexibility of satellite-borne network services. In the satellite environment, the rapid movement of the satellite nodes leads to rapid network changes, which presents the need for dynamic migration of different satellite nodes’ network functions on demand and in time to ensure the overall performance of the whole network. In this section, we propose a fast adaptive migration algorithm for network functions based on multidimensional environment awareness, and incorporate migration precomputation in collaboration with real-time computation to reduce the real-time computational overhead of network migration.

The scenario studied in this section is to achieve fast network load balancing through reasonable selection of VNFs and destination nodes to be migrated, reduce the number of VNF migrations and total migration overhead in the system, and improve system stability under the highly dynamic changes in network topology in the satellite environment. To improve migration efficiency, migration precomputation is introduced. In VNF selection, a reasonable VNF to be migrated is selected by the RAIL resource-aware algorithm; in destination node selection, a multi-objective decision is made by the TOPSIS multi-objective decision algorithm to reduce the additional delay brought by migration and the impact from migration for the destination node; through the algorithm for reasonable VNF selection and destination node selection, network function migration is carried out to achieve network load balancing.

### 4.1. VNF Migration Model and Optimization Problem

The VNF migration problem in NFV networks is presented in this subsection, and a network model is created to address it. This model describes the dynamic changes in demand in NFV networks, the occupancy of VNFs on different types of node resources, and the circumstances that lead to VNF migration. To lessen the pressure on the controller for the SFC end-to-end performance limits in VNF migration, equivalent conversion is carried out.

### 4.2. Network Model

For NFV networks $G=\left(V^{Node},E\right)$, $V^{Node}$ denotes the collection of nodes, and E represents the collection of links between nodes. The characteristics of the nodes and links are described as follows:

$N_{w}^{core}$: denotes the computing resources owned by node w, expressed as the number of cores.
$N_{w}^{mem}$: denotes the storage resources owned by node w, expressed in gigabytes.
$C_{i,j}$: denotes the link bandwidth between node i and node j. $i,j\in V^{Node}$.

$V^{VNF}$: denotes the collection of virtual network functions (VNFs), which are characterized as follows:

$n_{y}^{core}$: denotes the necessary computing resources for the virtual network operation y.
$n_{y}^{mem}$: denotes the necessary storage resources for the virtual network operation y.
$c_{y}$: denotes the necessary web resources for the virtual network operation y.
$a_{ij}$: denotes whether virtual network function $v_{i}$ is deployed on node $n_{j}$. $a_{ij}=1$ means $v_{i}$ is deployed on $n_{j}$, otherwise 0.

We define the utilization rate of a resource of a node as the ratio of the used resources to the total resources on the current node. The temporary condition for migration calculation is to perform the migration calculation immediately after any resource utilization rate of any resource exceeds its preset threshold $T_{k}$, $T_{k}\in \left(0,1\right),k=1,2,3$, and $T_{k}$ can be different for each resource.

For node $w_{j}$, the occupancy rate of its computing resources is $\frac{\sum_{i\in V^{VNF}}n_{i}^{core}•a_{ij}}{N_{w_{j}}^{core}}$, the occupancy rate of its storage resources is $\frac{\sum_{i\in V^{VNF}}n_{i}^{mem}•a_{ij}}{N_{w_{j}}^{mem}}$, and the occupancy rate of its network resources is $\frac{\sum_{i\in V^{VNF}}c_{i}•a_{ij}}{\sum_{k\neq j,k\in V^{Node}}C_{j,k}}$. The optimization goal is to minimize the overall system downtime by reasonably selecting the migrated virtual network function and the destination node for migration.

The set of service function chains (SFC) is F, $F=\left\{f_{1},f_{2},\ldots ,f_{n}\right\}$, and the maximum delay constraint for each function chain $f_{n}$ is $\tau_{n}$. Therefore, when the VNF to be migrated belongs to $f_{n}$ (assuming per VNF instance only belonging to one SFC), the sum of its additional delay $\tau_{+}$ and the original delay $\tau_{0}$ should be less than $\tau_{n}$, i.e., $\tau_{+}+\tau_{0}<\tau_{n}$.

The migration cost is defined as a function $F\left(cost,times\right)$ of the migration overhead $f\left(cost\right)$ and the number of migrations $f\left(times\right)$, i.e.,

(1) $$F\left(cost,times\right)=\lambda \cdot f\left(cost\right)+\mu \cdot f\left(times\right)$$

where λ and μ are adjustable weighting factors.

The migration overhead $f\left(cost\right)$ is the sum of VNF migration computation time and implementation migration time, and the number of migrations $f\left(times\right)$ is the number of VNF migrations that occur during the system operation. The migration computation time is determined by the number of system nodes and the performance of the controller; the implementation migration time is defined as the decommissioning time of the VNF that implements the migration, and the decommissioning time of $v_{i}$, i.e., the migration time $T\left(v_{i},n_{j},n_{k}\right)$ for $v_{i}$ to be migrated from node $n_{j}$ to node $n_{k}$ is:

(2) $$T\left(v_{i},n_{j},n_{k}\right)=\frac{n_{v_{i}}^{mem}}{B\left(v_{i}\right)}$$

where $n_{v_{i}}^{mem}$ denotes the size of storage resources occupied by virtual function $v_{i}$, and $B\left(f_{l}\right)$ is the transmission bandwidth occupied by virtual network function $v_{i}$ during migration.

#### 4.2.1. Optimization Problem

This section develops a multi-objective optimization problem to lower the overhead of VNF migration and the number of VNF migrations in NFV networks and to increase system stability. To lower the overhead of real-time computation during VNF migration in the system, a method of migration precomputation in collaboration with real-time computation is proposed.

#### 4.2.2. System Modeling

In order to achieve dynamic load balancing of the network and reduce system migration costs while still meeting user needs, migration cost is defined in this study as a function of migration overhead and the number of migrations.

(3) $$\min\;\left\{\lambda \cdot f\left(cost\right)+\mu \cdot f\left(times\right)\right\}$$

(4) $$subject\;to:\;\sum a_{ij}=1\;a_{ij}\in \left\{0,1\right\}$$

(5) $$\frac{\sum_{i\in V^{VNF}}n_{i}^{core}\cdot a_{ij}}{N_{w_{j}}^{core}}<T_{1},\;\;\forall w\in V^{Node}$$

(6) $$\frac{\sum_{i\in V^{VNF}}n_{i}^{mem}\cdot a_{ij}}{N_{w_{j}}^{mem}}<T_{2},\;\;\forall w\in V^{Node}$$

(7) $$\frac{\sum_{i\in V^{VNF}}c_{i}\cdot a_{ij}}{\sum_{k\neq j,k\in V^{Node}}C_{jk}}<T_{3},\;\;\forall w\in V^{Node}\;$$

(8) $$\sum_{l\in V^{VNF}}n_{l}^{core}\cdot a_{lk}+n_{i}^{core}\cdot b_{ik}<T_{1}\cdot N_{w_{k}}^{core}$$

(9) $$\sum_{l\in V^{VNF}}n_{l}^{mem}\cdot a_{lk}+n_{i}^{mem}\cdot b_{ik}<T_{2}\cdot N_{w_{k}}^{mem}$$

(10) $$\sum_{l\in V^{VNF}}c_{l}\cdot a_{lk}+c_{i}\cdot b_{ik}<T_{3}\cdot \sum_{k\neq j,l\in V^{VNF}}C_{jk}$$

(11) $$a_{ij}\cdot b_{ik}\cdot \Delta \tau_{jk+}+\tau_{0}<\tau_{max},v_{i}\in f_{n}$$

where $T_{1},\;T_{2},\;\;T_{3}$ denote the occupancy thresholds of computing, storage and network resources in the network nodes, respectively, and the migration computation is initiated right away if the usage rate of any resource exceeds its predetermined threshold $T_{k},\;\;T_{k}\in \left(0,1\right),k=1,2,3$. Each resource has a different $T_{k}$*,* which is determined by the system’s demand. $\tau_{max}$ denotes the maximum latency limit of the SFC to which the migrated $v_{i}$ belongs. Since the VNF to be migrated can be used by multiple service chains, the different SFCs have different requirements for latency, so we need to ensure that the latency of the VNF to be migrated is less than the minimum of the maximum latency received in all service chains to which the VNF belongs; the Boolean variable $b_{ik}=\left\{0,1\right\}$ denotes whether the virtual network function $v_{i}$ is moved to network node $n_{k}$. A flag variable $b_{ik}$ of 1 indicates that the virtual network function $v_{i}$ is migrated to node $n_{k}$, otherwise it is 0.

The objective Function (3) represents the migration cost and is a function related to the migration overhead and the number of migrations, where λ and μ are adjustable weighting factors. Constraint (4) guarantees that each virtual network function must and will be deployed on only one network node. Constraints (5)–(7) guarantee that the occupancy rate of computing, storage, and network resources of each network node will not exceed its pre-defined threshold. Constraints (8)–(10) ensure that the occupancy rate of computing, storage, and network resources of the destination network node will not exceed its pre-defined threshold after the virtual network function is migrated. Constraint (11) ensures that the sum of the additional delay due to virtual function migration and the original delay is less than the upper limit $\tau_{max}$ of the delay of the service function chain to which it belongs.

#### 4.2.3. Migration Precomputing and Real-Time Computing Synergy

The main migration overhead in VNF migration comprises the time overhead of calculating the VNF migration scheme and the time overhead of implementing the VNF migration scheme. When the number of nodes increases, the time overhead of performing migration scheme calculations increases sharply. To reduce the time overhead of migration scheme calculations, this paper proposes a migration algorithm that collaborates with migration precomputation and real-time computation, and sets the corresponding light overload threshold and overload threshold for the system to reduce the overhead of VNF real-time computation. To ensure the validity of the migration precomputation results, a time stamp is added to the migration computation results, and a reasonable timeout expiration time is set. In addition, the migration computation is performed again when the migration scheme in the result set is invalid after the timeout.

The implementation process of the migration algorithm with collaborative migration precomputation and real-time computation is as follows: the resource occupation of the node is monitored. When the node is overloaded, the node migration scheme result set is checked. If it is not empty and the expiration time has not expired, migration is performed, the node computation result set is set to empty immediately, and the node state continues to be monitored; if the node is overloaded and the node migration scheme result set is empty or non-empty, but the expiration time has expired, then the responsive migration strategy is used, the migration scheme is calculated in real time, and migration is implemented; when the node exceeds the light overload threshold but not the overload threshold, the node migration scheme result set is checked, and if the node migration scheme result set is empty or the expiration time has expired, the node migration scheme is re-calculated to ensure the validity of the migration scheme in the migration result set.

The system uses the same migration algorithm for precomputation and real-time computation, and the parameters that the algorithm receives include the node’s state, the occupancy information of the resources of the node that the VNF is installed on, and the pertinent state information of other nodes in the system.

#### 4.2.4. Selection Algorithm for the VNF to Be Migrated

In this paper, we introduce a resource-aware RAIL-based algorithm [21] for decision-making regarding the VNFs to be migrated.

When a node is overloaded and a VNF has to be migrated, the VNF to be migrated must be chosen based on the type of overloaded resource in order to reduce the resource occupancy of the overloaded node to a reasonable range as quickly as possible. The occupancy rate of each VNF on the node for each resource can be calculated as described in the previous section. We also defined the threshold value for each resource and defined a triplet $\left(\alpha ,\beta ,\gamma \right)$ to represent the overload status of the three resources of the node. When the computing resource is overloaded, α is assigned a value of 1. When it is not overloaded, α is assigned a value of 0, and similarly for the storage and network resources; the occupancy rate of each VNF on the node for each resource can be calculated as $\frac{n_{i}^{core}}{N_{w_{j}}^{core}}$, $\frac{n_{i}^{mem}}{N_{w_{j}}^{mem}}$, $\frac{c_{i}}{\sum_{k\neq j,k\in V^{Node}}C_{j,k}}$.

We define the migration index $\theta_{i}$, add the perception of a multidimensional environment, and use the RAIL dynamic weight setting to ensure that the weight of overloaded resources is greater than 1, the weight of lightly loaded resources is less than 1 according to the usage of corresponding resources, and the migration index of virtual functions with more usage of overloaded resources is greater.

(12) $$\theta_{i}=\sum_{K}\left\{\alpha_{k}\frac{1}{1-x_{k}}+\left(1-\alpha_{k}\right)\left(1-x_{k}\right)\right\}\chi_{ik},\;K=\left(Core,Mem,Com\right)$$

where $\alpha_{k}$ indicates whether the resource type *k* is overloaded, $x_{k}$ indicates the node’s resource type *k*’s occupancy, and $\chi_{ik}$ denotes the occupancy of $v_{i}$ on the resource type *k* of the node.

All VNFs on the overloaded nodes are ranked according to the migration index, and the VNF with the highest migration index is chosen to enter the VNF migration sequence.

#### 4.2.5. Migration Destination Node Selection Algorithm

The performance needs of the VNF to be migrated and the end-to-end performance limitations of the functional service chain in which the VNF is placed must both be taken into account when choosing the migration destination node. When the constraints are met, the destination node is chosen based on which node has the most residual resources and the least amount of delay. The TOPSIS algorithm is used to choose the destination node since choosing the best node entails making a multi-objective decision.

In the first step, nodes whose additional latency meets the constraints are selected according to the end-to-end performance requirements of the functional chain of the service in which the VNF is to be migrated.

(13) $$a_{ij}\cdot b_{ik}\cdot \Delta \tau_{jk+}+\tau_{0}<\tau_{\max},v_{i}\in f_{n}$$

In the second step, the node that meets the performance requirements of the VNF and will not be overloaded after the VNF moves in is selected from among the nodes selected according to their performance requirements.

In the third step, the TOPSIS algorithm is used to calculate the migration index of the nodes [22].

First, the positive ideal solution BestNode is calculated, where each property of the positive ideal solution is the best value among all alternatives.

(14) $$T_{i+}=\min\left\{T_{ki}|k\in N_{3}\right\},i\in \left\{1,2,3\right\}$$

(15) $$\tau_{+}=\min\left\{\tau_{k}|k\in N_{3}\right\}$$

The minimum utilization of each resource in all of the nodes to be selected as the occupancy rate of the ideal solution $\left(T_{1+},T_{2+},T_{3+}\right)$ is calculated; at the same time, it is also desired to optimize the additional delay caused by the migration, to derive the minimum additional delay $\tau_{+}$ for all of the nodes to be selected as the additional delay caused by the migration of the ideal virtual network function.

The negative ideal solution Worst Node is calculated, where each property of the negative ideal solution is the worst value among all alternatives.

(16) $$T_{i-}=\max\left\{T_{ki}|k\in N_{3}\right\},i\in \left\{1,2,3\right\}$$

(17) $$\tau_{-}=\max\left\{\tau_{k}|k\in N_{3}\right\}$$

The maximum utilization of each resource in all nodes to be selected as the occupancy of the negative ideal solution $\left(T_{1-},T_{2-},T_{3-}\right)$ is calculated, while the additional time delay of the negative ideal solution is $\tau_{-}$.

The Euclidean distance $\theta_{ok+}$ of the node $w_{k}$ from the BestNode and the Euclidean distance $\theta_{ok-}$ from the WorstNode are calculated, and the proximity $\theta_{ok}$ of the node to the BestNode is obtained.

(18) $$\theta_{ok+}=\sqrt{\sum_{i=1}^{3}{\left[\gamma_{i}\left(T_{ki}-T_{i+}\right)\right]}^{2}+{\left[\gamma_{4}\left(\tau_{k}-\tau_{+}\right)\right]}^{2}}$$

(19) $$\theta_{ok-}=\sqrt{\sum_{i=1}^{3}{\left[\gamma_{i}\left(T_{ki}-T_{i-}\right)\right]}^{2}+{\left[\gamma_{4}\left(\tau_{k}-\tau_{-}\right)\right]}^{2}}$$

(20) $$\theta_{ok}=\frac{\theta_{ok-}}{\theta_{ok-}+\theta_{ok+}}$$

where $\gamma_{i}$ denotes the weight of each indicator and is a predefined value; $\theta_{ok}$ denotes the proximity of the node to the positive ideal solution, with larger $\theta_{ok}$ indicating that the node is closer to the ideal solution.

According to the migration index $\theta_{ok}$ of each node, the one with the largest migration index is selected as the migration point.

## 5. Simulation and Performance Analysis

### 5.1. Performance Evaluation

**Figure 4:**
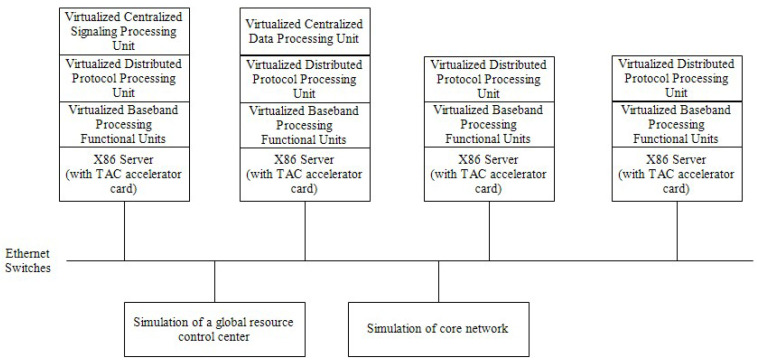
Schematic diagram of the validation environment for the digital satellite-borne base station system.

The digital satellite-borne base station system’s validation environment was constructed as depicted in Figure 4. A high-performance X86 server and a TAC accelerator card with a high-performance FPGA made up the hardware component for simulating the single satellite function, with the X86 server handling the simulation of the satellite-borne protocol processing unit and the TAC accelerator card handling the simulation of the satellite-borne baseband processing unit. The verification environment was composed of the process resource for four satellites and one global resource control center that was imitated by a server and one core network that was simulated by a PC. Each processing unit had a high-orbit satellite communication system communication protocol and baseband software loaded through the global resource control center. The high-orbit satellite communication system was composed of communication protocol software and baseband software, which was developed according to the standard GMR-1 3G from ETSI. The protocol software performed the L2-L3 layer’s functions according to the standard, and the baseband software performed the high-PHY functions, which included only the control flow in PHY but not the signal processing. The RF path and feeder link components were streamlined and realized through an Ethernet connection in order to simplify the environment building task. To virtualize the functions of baseband software processing and protocol software processing, the virtualization platform software had to first be loaded on each server. For better virtualization, the protocol software was split into three parts, which were distributed protocol processing unit software, centralized signaling processing unit software and centralized data processing unit software. Then, through the global resource control center, a high-orbit satellite communication system virtualized baseband processing function unit software program was loaded on a virtualization platform of four servers, a high-orbit satellite communication system virtualized distributed protocol processing unit software program was loaded on the virtualization platform of four servers, and a high-orbit satellite communication system virtualized centralized signaling processing unit was loaded on the virtualization platform of one of the servers according to the functional requirements. The centralized signaling processing unit was loaded on the virtualization platform of one server, and the virtualized centralized data processing unit of a high-orbit satellite communication system was loaded on the virtualization platform of one server. These four servers were identical in terms of their hardware configuration, but the dynamic reconfiguration and flexible networking of each satellite protocol function were achieved by simulating the scheduling management, dynamic loading, and configuration management of baseband resources and protocol processing resources in the global resource control center.

Since there was no precedent for a base station on a satellite, this validation system was compared with the deployment of an existing high-orbit satellite mobile communication system in terms of performance. The existing high-orbit satellite mobile communication system was composed of dedicated equipment that met the ATCA standard with a different functional hardware board.

In order to compare the performance between the digital satellite-borne base station system and the existing high-orbit satellite base station system, four experiments were performed. First of all, the single functional unit deployment time was considered. The single functional unit deployment time was defined as the time for adding only one functional unit when the system is running (e.g., protocol processing unit), and the steps for the two systems were as below. 

For the traditional ground base station:

(1) Picking a dedicated protocol hardware board.
(2) Uploading the protocol software and performing some configuration on the board.
(3) Connecting the board to the system, and performing some configuration on the other board to attach the new protocol hardware board.
(4) Powering up the new board and waiting for the board startup.

For the digital satellite-borne base station:

(1) Typing commands on the global resource control center (a PC) that include bringing up the virtual machine and uploading the configuration file and images.
(2) Waiting for the new software startup.

Secondly, the minimum system deployment time for network elements was considered and defined as the time for setup of a new base station including all of the functions.

For the traditional ground base station:

(1) Preparing all of the functional hardware boards.
(2) Uploading the relevant software on each board and performing some configuration on each board.
(3) Connecting all of the boards and performing some configuration.
(4) Powering up the system and waiting for the board startup.

For the digital satellite-borne base station:

(1) Typing commands on the global resource control center (a PC) that include bringing up the virtual machine and uploading the configuration file and images.
(2) Waiting for the system startup.

Thirdly, the system software upgrade time is considered which is defined as the time for upgrade the whole system when the system is running.

For the traditional ground base station:

(1) Uploading every single software on each hardware board one by one.

It is much more complicated to upgrade the hardware. The system should be stopped and the hardware boards swapped. Once the configuration is complete, the system should be restarted.

For the digital satellite-borne base station:

(1) Typing commands on the global resource control center (a PC) that include uploading the configuration file and images.

To upgrade hardware, one can simply move the existing system to other hardware via the virtualization platform and swap out the old system, then move the system back in via a command from the global resource control center.

Performing these steps takes much more time on a traditional ground base station system.

Finally, we considered the single functional unit reconfiguration time, which was defined as the time for the reconfiguration of a single functional unit (e.g., the centralized signal processing unit).

Because each board had its own software on the traditional ground base station, it was hard to split the software into much smaller components. Software cannot be reconfigured for only a single functional unit; this can be achieved only on a digital satellite-borne base station system.

Table 2 shows the results of the comparison between a traditional ground base station system and digital satellite-borne base station system in terms of single functional unit deployment time, minimum system deployment time, system software upgrade time, and single functional unit reconfiguration time. Because a traditional ground base station system with dedicated devices needs to deploy every functional element with a specific device and specific mechanism for installing software, whereas a digital satellite-borne base station system deploys every functional element with the same mechanism for installing software on the same hardware platform, the single functional unit deployment time for a digital satellite-borne base station system is much lower than that of a traditional base station system. On a ground base station system, not only is the software installation complex, but different devices also need to be connected. Compared with a traditional ground base station system, the deployment can be completed by directly configuring the software on the digital satellite-borne base station system. Therefore, the minimum system deployment time for a traditional ground station system is much longer than that of a digital satellite-borne base station system. As a digital satellite-borne base station system uses a unified software installation method, compared with the independent installation tasks associated with each component of a traditional ground station system, its upgrade time will also be greatly improved. Because of the flexible architecture of a digital satellite-borne base station system, it is possible to reconstruct a single functional unit in time as needed. That possibility is not available with a traditional ground station system.

As can be seen, the system mentioned in this paper outperformed the first-generation high-orbit satellite signaling gateway station system in terms of single-function unit deployment time, minimum system deployment time, and system software upgrade time by at least 30-fold, 120-fold, and 240-fold, respectively, and could achieve a breakthrough in reconfiguration of unit functions from scratch. The digitized satellite base station system proposed in this research increases the adaptability of the satellite network service and enables rapid network deployment, flexible scheduling, and dynamic function loading.

### 5.2. Migration Algorithm Performance Evaluation

The network was initially configured with 100 nodes and 500 randomly generated links, which was around the size of a medium-sized ISP network [23]. Each node was given 10 normalized units of storage, computing, and networking resources, plus a random value between 1 and 10 units for inter-link delay. The service traffic in the network was random, so the corresponding virtual network functions’ occupancy of resources was dynamically changing. It was assumed that each network service function chain consisted of two to five virtual network functions, one to five virtual network functions placed on each node, and a certain number of idle nodes, i.e., nodes where no virtual network functions were placed, added to the network to improve the stability of the system.

The migration of a node in the network was determined by a migration trigger threshold and triggered when any resource utilization rate of the node that the virtual network function was on reached the threshold. Contrary to conventional periodic migration, the adaptive nature of the approach used in the present study was reflected in this trigger threshold-determined migration. The migration cost was defined as a function of the migration overhead and the number of migrations, where the migration overhead was the sum of the time spent on VNF migration calculation and the time spent on migration implementation, and the number of migrations was the number of VNF migrations that occurred during system operation; in the simulation experiments, it was assumed that the demand in the network was updated every second, and the VNF resource occupation on the corresponding node changed accordingly, and after 24 h of continuous updates, the migration overhead per unit time and the number of migrations per unit time were calculated to compare the performance of each algorithm.

Table 3 shows a detailed comparison of the similarities, differences and expected benefits of the four experimental methods.

The first method used the RAIL&TOPSIS cooperative resource-aware algorithm. To find the best migration solution using a combination of the two methods, the VNFs to be migrated were chosen to use the RAIL algorithm for resource awareness, and the overloaded nodes most in need of VNF migration were chosen to be added to the VNF migration sequence through the migration index, and the ideal destination nodes were chosen to use the TOPSIS multi-objective decision-making algorithm , but this algorithm would increase the computational complexity and migration computation time. The second algorithm was the fixed weight method; this algorithm uses fixed weights in calculating the VNF to be migrated. The third algorithm was the simple quick migration algorithm, which simplifies the computational complexity and accelerates the migration speed in the migration destination node selection stage, and uses the node as the migration destination node when the performance and delay constraints are satisfied. The fourth algorithm combined the pre-calculation RAIL and TOPSIS algorithms to reduce the solution time and migration overhead by adding pre-calculation in collaboration with real-time computation.

**Figure 5:**
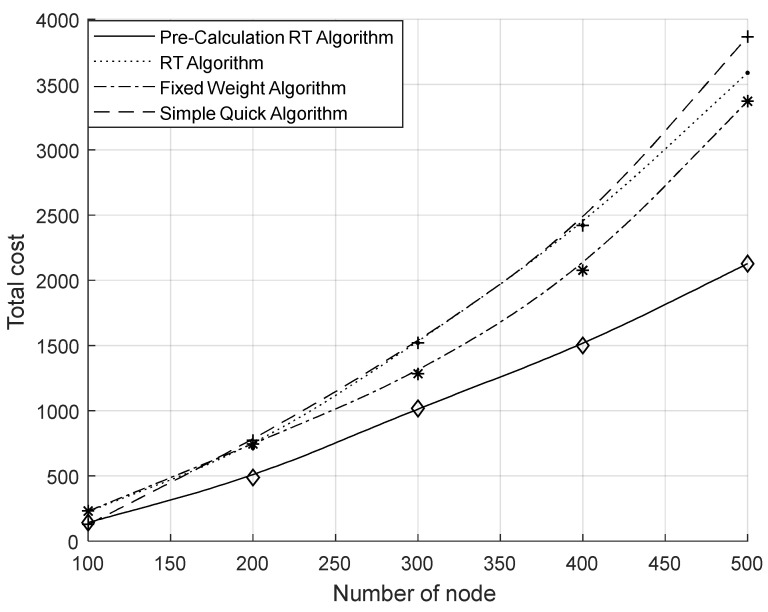
Comparison of total migration overhead.

**Figure 6:**
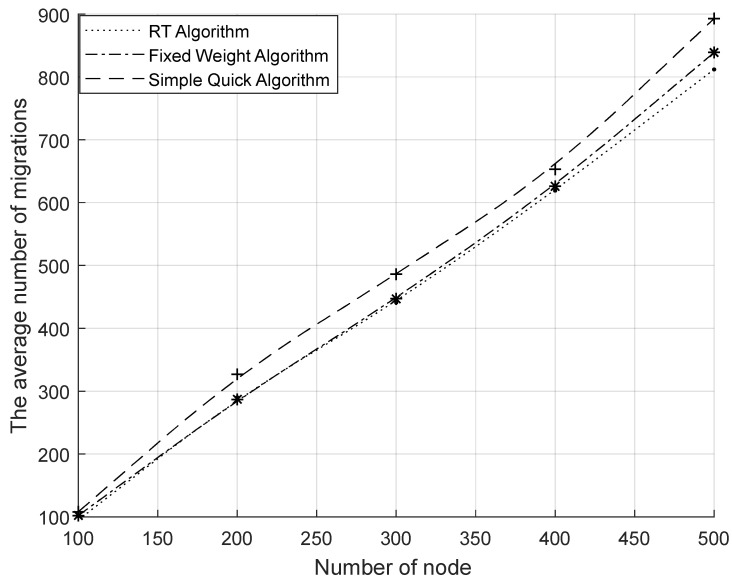
Comparison of the average number of migrations.

**Figure 7:**
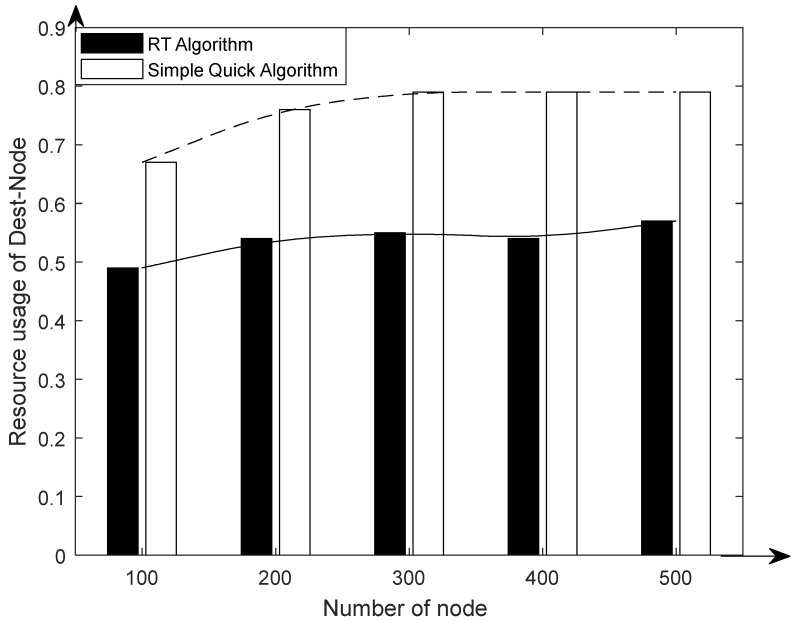
Comparison of resource usage of migration destination nodes.

**Figure 8:**
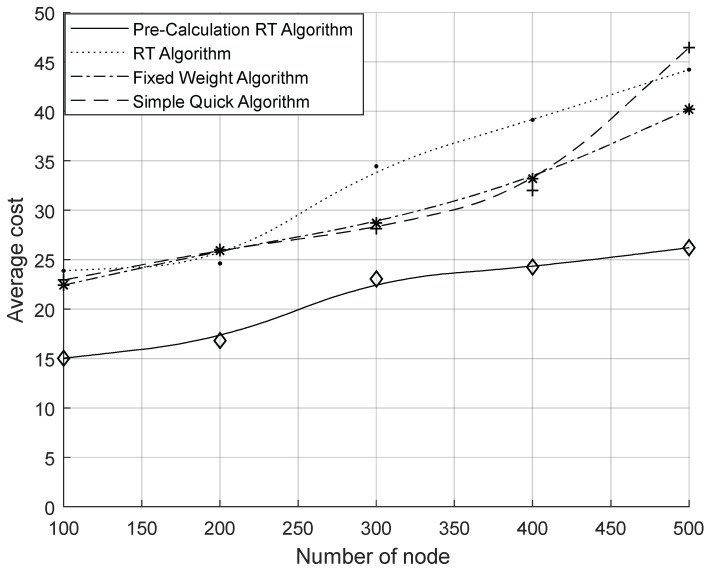
Comparison of average single migration costs.

Figure 5 shows the comparison of the total migration cost of the system under different migration algorithms. From the figure, it can be seen that the total migration cost of the system increased as the node size increased; the RT algorithm had a higher total migration cost than other algorithms because of its higher computational complexity. However, with the precomputation process included, the system’s overall migration cost was considerably less than that of the other two basic approaches. From Figure 6, it can be seen that the number of migrations per unit time of the system increased with the increase in the number of nodes. Compared with the other two algorithms, the number of migrations was significantly reduced by the RT algorithm. Comparing the RT algorithm with the fixed weight algorithm, it can be concluded that the sensing of the RAIL algorithm can effectively reduce the number of migrations. Comparing the RT algorithm with the simple instant algorithm, it was found that the multi-objective decision-making of TOPSIS could effectively reduce the number of migrations. Figure 7 shows the average resource occupancy of the destination nodes when the RT algorithm and the simple instant algorithm were used to select the destination nodes for migration. This figure shows that for various numbers of nodes, the RT method used less of the destination node’s resources, on average, compared to the simple instantaneous approach. Figure 8 shows the single migration cost of a VNF under different algorithms. From the figure, it can be seen that the single migration cost based on the RT algorithm was significantly higher than that of other algorithms because of its higher migration overhead and lower number of migrations.

In summary, the RT algorithm can effectively reduce the number of migrations and achieve more efficient network balancing, but it brings higher computational complexity and increases the migration computation time, resulting in higher single migration costs. To retain the advantages of the RT algorithm and reduce the migration computation time, we propose the RAIL&TOPSIS algorithm (PRT algorithm) with the collaboration of precomputation and real-time computation by adding the precomputation mechanism to the RT algorithm. Figure 5 shows that following the addition of precomputation, a significant reduction was achieved in the system’s overall migration costs, which were lower than those of the fixed weight algorithm and the simple instantaneous algorithm. Figure 8 shows a comparison between the PRT algorithm and the RT algorithm, which effectively lowered the cost of a single migration to levels s lower than those of the other two baseline methods. Because the number of migrations was the same as that of the RT algorithm, the PRT algorithm with its precomputation mechanism could effectively reduce its migration computation time, resulting in a reduction in migration costs. At the same time, because the number of migrations was the same as that of the RT algorithm, the cost of a single migration was also significantly lower than that of other algorithms.

## 6. Conclusions

The virtualization of the structure of a satellite-borne base station system and the transformation of hardware products to firmware and software applications have made the structure of the satellite infrastructure simple and cost-effective, and the service provision more dynamic and efficient, enabling satellite network operators to achieve rapid demand fulfillment, flexible resource scheduling, effective cost control, significant performance improvements and green network intensification. This paper proposes an architecture for a horizontal shared virtual network that converges computing, communication, and storage. Through the constructed verification environment and comparison between a traditional satellite base station system and the virtualization satellite-borne base station in terms of deployment time and reconfiguration time, it was shown that the virtualization architecture had big advantages in flexibility and reconfigurability of the satellite-borne base station system.

This paper also proposes a VNF multi-domain resource-aware migration algorithm based on the collaboration of migration precomputation and real-time computation, which senses the resource occupation of overloaded nodes by the RAIL algorithm and selects VNFs to migrate out. Based on the resource requirements of the VNF and the end-to-end performance constraints of the SFC, TOPSIS was used for multi-objective decision-making to achieve better network balancing. This algorithm realized the rapid deployment of a digital satellite-borne base station system network and improved the stability of the system in the satellite environment.

The simulation results demonstrate that the VNF multi-domain resource-aware migration algorithm using migration precomputation in conjunction with real-time computation can improve system stability while minimizing migration overhead, select the best migration destination nodes, reduce the overall number of system migrations, and improve the stability of the system.

After an architecture of a horizontal shared virtual network has been designed and also the problem of VNF migration time under this architecture is fixed, it will be possible to deploy network functions on an LEO satellite as we wish. So, future work on how to deploy the network function appropriately to maximize the performance of the system should be conducted. For example, the questions of to optimize the design and deploy the network function (NF) on an LEO satellite according to the LEO satellite dynamics and communication requirements to achieve the goal of reducing signaling transmission time should be solved. This will enable finding the most appropriate service function chain (SFC) for users in an LEO satellite constellation. Based on the architecture to improve the performance of the LEO satellite system, other system indicators also can be optimized, except for the signal transmission time.

## Figures and Tables

**Table 1:** Architecture Comparison.

Architecture	Where to Process Signal and Data	Transmission Delay	Deployment Time	Considers Limitations of Satellite Resources
MEO satellites [2] and 5G NTN [3]	Processing by ground station	Long	Long	No
IoT in future NTN [5]	Processing on satellite	Short	Short	No
Satellite–ground network for 6G [4] ※	Processing on satellite	Short	Short	Yes
Architecture proposed in this paper	Processing on satellite	Short	Short	Yes

※ This is a review article.

**Table 2:** Deployment Performance Comparison.

Compared Items	An Existing High-Orbit Satellite Base Station System	Digital Satellite-Borne Base Station System
Single functional unit deployment time	≥30 min	≤60 s
Minimum system deployment time for network elements	≥4 h	≤120 s
System software upgrade time	≥4 h	≤60 s
Single functional unit reconfiguration time	Not functional	≤60 s

**Table 3:** Comparison of Algorithmic Similarities, Differences and Expected Benefits.

Algorithm	Similarities with RT Algorithm	Differences from the RT Algorithm	Expected Benefits
RT algorithm (RAIL&TOPSIS)	— — —	— — —	Obtaining the best migration solution under the optimization goal, but the complexity of the migration calculation is too high.
Fixed weight algorithm	The best destination node is selected using TOPSIS multi-objective decision-making algorithm.	The VNF to be migrated is chosen to be legacy and not resource-aware.	Reduces computational complexity to some extent, but does not speed up node recovery rate.
Simple quick algorithm	VNFs to be migrated are chosen to adopt RAIL for resource awareness.	The destination node is selected using the performance constraints that are satisfied by that node.	Reduces the computational complexity of migration, but increases the number of migrations and reduces the stability of the system.
PRT algorithm	The RAIL and TOPSIS algorithms are also used.	Incorporates a pre-computation mechanism into the migration process to speed up the rate of migration.	Greatly reduces the overhead of real-time migration calculations during migration.

## Data Availability

Not applicable.
